# Supplementary material for: Structural Insights and Calcium-Switching Mechanism of Fasciola hepatica Calcium-Binding Protein FhCaBP4
Source: Int J Mol Sci. 2025 Aug 5;26(15):7584. doi: 10.3390/ijms26157584 (PMC12347358; doi:10.3390/ijms26157584)
Supplement: Supplementary file 1 [file ijms-26-07584-s001.zip › ijms-3780496-supplementary.pdf]

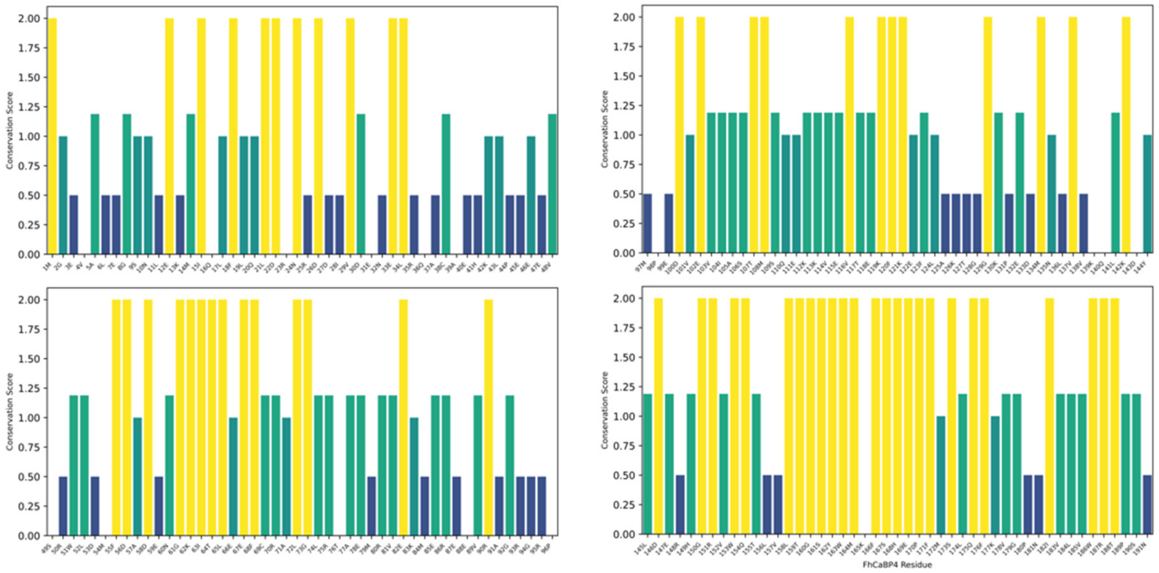

Supplementary Figure S1. Sequences conservation scores for FhCaBP4. Shannon entropy was computed at each alignment position, excluding gaps, and conservation scores were visualized as bar plots to identify highly conserved residues across the sequences

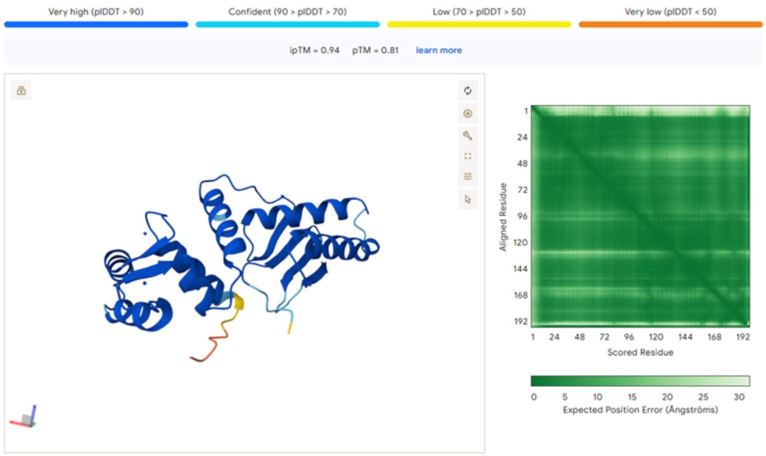

Supplementary Figure S2. AlphaFold3 prediction of FhCaBP4. Cartoon representation coloured by per-residue prediction confidence (pLDDT): dark blue > 90, light blue 70–90, yellow 50–70, orange < 50. Predicted aligned error (PAE) matrix for all residue pairs; dark green indicates 0 Å expected error, shading to white at 30 Å. Global scores (ipTM = 0.94; pTM = 0.81) are shown above the panel.

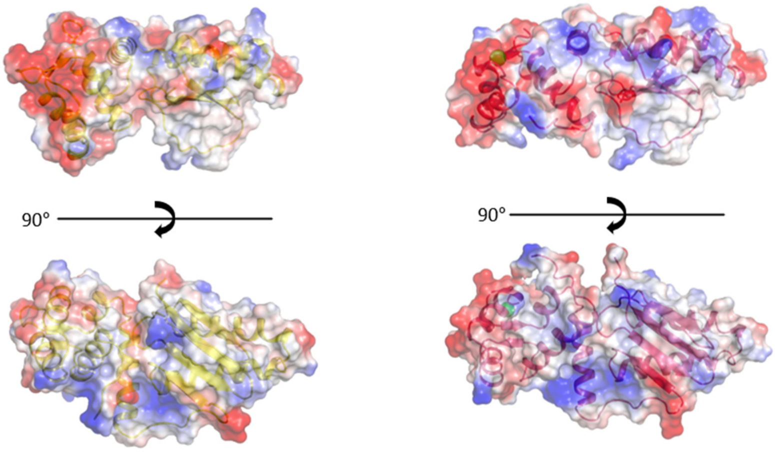

Supplementary Figure S3. Electrostatic surfaces of the FhCaBP4 Apo structure (yellow) and predicted calcium bound FhCaBP4 model (pink).

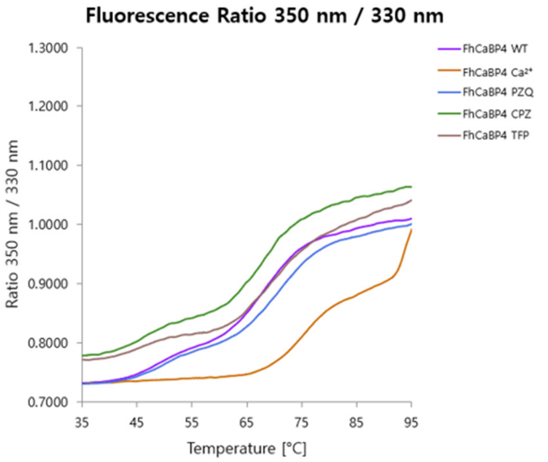

Supplementary Figure S4. Thermal unfolding curves. Representative unfolding profile curves of FhCaBP4 WT, FhCaBP4 + Calcium, FhCaBP4 + PZQ, FhCaBP4 + CPZ, and FhCaBP4 + TFP obtained by intrinsic fluorescence measurements by using Tycho NT. 6.
